# The complete mitochondrial genome of *Haemaphysalis concinna* (Ixodida: Ixodidae)

**DOI:** 10.1080/23802359.2018.1450669

**Published:** 2018-03-14

**Authors:** Qiao-Cheng Chang, Xue Fu, Chun-Ling Song, Hong-Bo Liu, Yi Sun, Na Jia, Jia-Fu Jiang, Chun-Ren Wang, Bao-Gui Jiang

**Affiliations:** a State Key Laboratory of Pathogen and Biosecurity, Beijing Institute of Microbiology and Epidemiology, Beijing, PR China;; b College of Animal Science and Veterinary Medicine, Heilongjiang Bayi Agricultural University, Daqing, Heilongjiang, PR China

**Keywords:** *Haemaphysalis concinna*, mitochondrial genome, phylogenetic analysis

## Abstract

The complete mitochondrial genome of *Haemaphysalis concinna* is reported for the first time in this study. Its entire mitogenome is 14,675 bp in length, contained 13 protein-coding genes, two ribosomal RNA genes, 22 transfer RNA genes, and two non-coding regions. Among the 13 protein-coding genes, apart from the *nad*1, *nad*4, *nad*4L, and *nad*5 gene encoded on the L-strand, the remaining protein-coding genes were encoded on the H-strand. The phylogenetic analysis by Bayesian inference method shows that *Amblyomma sphenodonti* and *H. parva* formed one clade, while *H. concinna* and other species of genus *Haemaphysalis* formed the other clade, indicating that *H. concinna* belong to the genus *Haemaphysalis*.

The obligate haematophagous ectoparasites *Haemaphysalis concinna* (Ixodida: Ixodidae) is widely distributed in China (Teng and Jiang 1991), Russia, Germany, as well as temperate Eurasia (Nosek 1971). These adult stages of *H. concinna* mainly parasitize on Artiodactyla, and accidently attack humans that cause mechanical damage and transmit a great variety of pathogens (Mikryukova et al. 2014; Švehlová et al. 2014).

The adult of *H. concinna* was collected by swiping flags on vegetations from Seven peak national Forest Park (46.711100 N, 130.945300 E, 550 m at attitude), Huanan County Heilongjiang Province in Northeast China, on 12 April 2016. The individual tick was stored in the Department of Parasitology, Heilongjiang Bayi Agricultural University (specimen no. BYNKPL-160412), and DNA was extracted by TIANamp Genomic DNA Kit (TIANGEN, Beijing, China), and stored at −20 °C until use. The entire mitochondrial genomic sequences were composed of two overlapping fragments, one fragment approximately 5.9 kb was amplified from *cox*1 to *rrn*L using the primers CO1-J (5′-CCT GAT ATA GCA TT TCC TCG-3′) and 16S-N (5′-CTG CTC AAT GAT TTT TAA ATT GCT GTG-3′), the other one fragment approximately 9.0 kb was amplified from *rrn*L to *cox*1 using the primers 16S-J (5′-TTA CGC TGT TAT CCC TAG AGT ATT-3′) and CO1-N (5′-GCT ATA TCA GGT GCA CCT-3′).

The entire *H. concinna* mt genome was a typical circular DNA molecule with 14,675 bp in size (GenBank accession number NC_034785), which contained 13 protein-coding genes (*cox*1-3, *nad*1-6, *nad*4L, *atp*6, *atp*8, and *cyt*b), two ribosomal RNA (rRNA) genes, 22 transfer RNA (tRNA) genes, and two non-coding regions (NCRs). The protein-coding genes were transcribed in different directions, which were consistent with those of other ticks (Black and Roehrdanz 1998; Burger et al. 2014; Guo et al. 2016), but distinct from those of trematodes, cestodes, and nematodes (Yamasaki et al. 2012; Duan et al. 2015; Chang et al. 2016), which transcribed in the same directions. Among the 13 protein-coding genes, apart from the *nad*1, *nad*4, *nad*4L, and *nad*5 gene encoded on the L-strand, the remaining protein-coding genes were encoded on the H-strand. The nucleotide compositions of the complete mtDNA sequence of *H. concinna* were biased towards A + T (77.96%), with T being the most favoured nucleotide (39.35%) and G was the least favoured (9.21%). The *H. concinna* mt genome encoded 3615 amino acids in total. The A + T content of protein-coding genes ranged from 71.02% (*cox*1) to 84.57% (*atp*8).

Based on the concatenated amino acid sequence dataset (13 protein-coding genes), phylogenetic analyses were performed using Bayesian inference (BI). The result showed that the tree was divided into two large branches: Prostriata and Metastriata (Figure 1). Within the metastriate, *H. concinna* and genus *Haemaphysalis* species clustered together with high statistical support (PP = 1), indicating that *H. concinna* belong to the genus *Haemaphysalis*. This study provides not only new mtDNA resource for phylogenetic studies, but also novel and useful genetic marker for further studies on species identification, population genetics, and molecular epidemiology of the genus *Haemaphysalis* in ticks.

**Figure 1.**
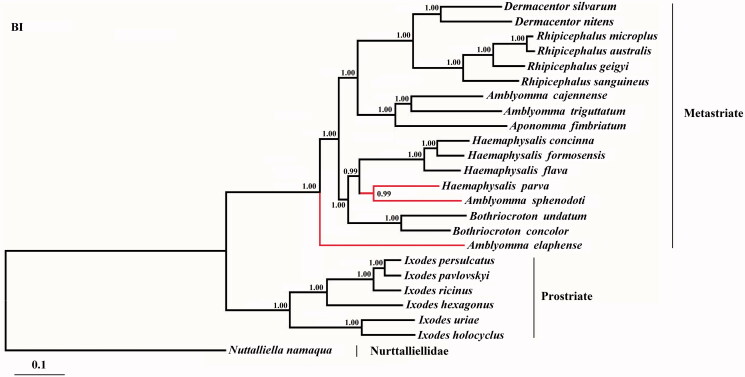
Phylogenetic relationships of *Haemaphysalis concinna* and other species based on mitochondrial sequence data. The concatenated amino acid sequences of 13 protein-coding genes were analysed with Bayesian inference (BI), using *Nuttalliellidae namaqua* (NC_019663) as an outgroup. All the species accession numbers in this study are listed as below: *Amblyomma triguttatum* NC_005963, *Amblyomma elaphense* NC_017758, *Amblyomma sphenodonti* NC_017745, *Amblyomma cajennense* NC_020333, *Aponomma fimbriatum* NC_017759, *Bothriocroton concolor* NC_017756, *Bothriocroton undatum* NC_017757, *Haemaphysalis flava* NC_005292, *Haemaphysalis formosensis* NC_020334, *Haemaphysalis parva* NC_020335, *Rhipicephalus sanguineus* NC_002074, *Rhipicephalus microplus* KP143546, *Rhipicephalus australis* NC_023348, *Rhipicephalus geigyi* NC_023350, *Ixodes hexagonus* NC_002010, *Ixodes holocyclus* NC_005293, *Ixodes persulcatus* NC_004370, *Ixodes uriae* NC_006078, *Ixodes pavlovskyi* NC_023831, *Ixodes ricinus* NC_018369, *Dermacentor nitens* NC_023349, *Dermacentor silvarum* NC_026552, and *Nuttalliella namaqua* NC_019663.
